# KCTD5 Forms Hetero-Oligomeric Complexes with Various Members of the KCTD Protein Family

**DOI:** 10.3390/ijms241814317

**Published:** 2023-09-20

**Authors:** Yini Liao, Douglas C. Sloan, Josephine H. Widjaja, Brian S. Muntean

**Affiliations:** Department of Pharmacology and Toxicology, Medical College of Georgia, Augusta University, Augusta, GA 30912, USA; yliao@augusta.edu (Y.L.); jwidjaja@augusta.edu (J.H.W.)

**Keywords:** Potassium Channel Tetramerization Domain (KCTD)-containing protein, KCTD2, KCTD5, KCTD17, KCTD8, KCTD12, KCTD16, hetero-oligomer

## Abstract

Potassium Channel Tetramerization Domain 5 (KCTD5) regulates diverse aspects of physiology, ranging from neuronal signaling to colorectal cancer. A key feature of KCTD5 is its self-assembly into multi-subunit oligomers that seemingly enables participation in an array of protein–protein interactions. KCTD5 has recently been reported to form hetero-oligomeric complexes with two similar KCTDs (KCTD2 and KCTD17). However, it is not known if KCTD5 forms hetero-oligomeric complexes with the remaining KCTD protein family which contains over two dozen members. Here, we demonstrate that KCTD5 interacts with various KCTD proteins when assayed through co-immunoprecipitation in lysed cells. We reinforced this dataset by examining KCTD5 interactions in a live-cell bioluminescence resonance energy transfer (BRET)-based approach. Finally, we developed an IP-luminescence approach to map regions on KCTD5 required for interaction with a selection of KCTD that have established roles in neuronal signaling. We report that different regions on KCTD5 are responsible for uniquely contributing to interactions with other KCTD proteins. While our results help unravel additional interaction partners for KCTD5, they also reveal additional complexities in KCTDs’ biology. Moreover, our findings also suggest that KCTD hetero-oligomeric interactions may occur throughout the KCTD family.

## 1. Introduction

The Potassium Channel Tetramerization Domain (KCTD) family contains more than two dozen proteins [1]. KCTD proteins are increasingly being recognized as prominent players in the pathophysiology of neurodevelopmental diseases, neuropsychiatric disorders, and oncogenic pathways in cancer [2,3]. Although the majority of KCTD proteins remain relatively uncharacterized, a common thread among this family is the ability to participate in networks of protein–protein interactions, which enables regulation of cellular signaling events [4,5,6,7,8,9]. Therefore, investigating the principles of KCTD protein interactions will greatly advance our understanding of their diverse physiological roles that underly disease states.

An integral feature of most KCTDs is their self-assembly into multi-subunit oligomers [10,11,12]. This contributes to KCTDs’ engagement with multiple proteins. Perhaps the most studied example pertains to KCTD12 regulation of GABA_B_-GIRK signaling [13]. GABA_B_ activates Gβγ association with a GIRK channel, whereby Gβγ removal desensitizes the signal. A pentameric KCTD12 complex binds the GABA_B_ receptor, which tethers KCTD subunits proximal to the GIRK channel to cooperatively sequester Gβγ subunits and regulate signaling [4]. Interestingly KCTD12 also forms a hetero-oligomeric complex with KCTD16, which further shapes the kinetics of GABA_B_-GIRK signaling [14]. Another example pertains to KCTD5, a Cullin3 (Cul3) ubiquitin ligase adapter [15], which forms a hetero-oligomeric complex with KCTD2 [16,17] and KCTD17 [16]. KCTD5 remains of further interest because it reportedly endows KCTD2 the ability to interact with Cul3 [17]. KCTD5 has also been observed to play a major role in regulating neuronal signaling through G protein-coupled receptors (GPCRs) [6,8] and promoting motor behavior in vivo [7]. This raises the possibility that KCTD5 may serve a broader role as a scaffold partner with other KCTD proteins. In the context of disease, KCTD5 has been identified as a novel cancer biomarker [18] that exhibits high expression in some lung adenocarcinoma tumors [19]. Intriguingly, the expression of other KCTD members decreased in the advanced tumor stage [19], suggesting that interplay between KCTDs may play a role in disease progression. Therefore our goal was to examine KCTD5 hetero-oligomer complex formation across the entire KCTD protein family.

Here, we investigated if KCTD5 interacts with KCTD proteins utilizing two cell-based approaches. Through cellular co-immunoprecipitation (Co-IP) and bioluminescence resonance energy transfer (BRET)-based experiments, we uncovered that KCTD5 interacts with multiple members of the KCTD family. We then utilized a novel IP-luminescence strategy to map which regions of KCTD5 are required for interaction with a selection of disparate KCTDs that prominently regulate neuronal signal transduction processes (KCTDs 2, 5, 8, 12, 16, and 17). Our results further suggest that KCTDs’ biology may be more broadly nuanced by increasing complexity in KCTD subunit configurations beyond KCTD5.

## 2. Results

### 2.1. Assaying KCTD5’s Interaction with KCTD Proteins

In order to profile the interactivity of KCTD5 with each individual member of the KCTD family, we first developed two cellular approaches utilizing HEK293 cells that would robustly report such interaction. To gauge the feasibility of such experiments, we first examined KCTD5’s interaction with itself, which has been observed by the fact that KCTD5 forms a pentameric ring structure [10]. Moreover, KCTD5 has a high degree of similarity with KCTD2 and has also been reported to interact with KCTD2 [17]. KCTD5 is also similar to KCTD17 and to a lesser extent KCTD9 [6]. Therefore we first probed KCTD5’s interaction with KCTD2, KCTD5, KCTD9, and KCTD17. The rationale was that KCTD5 would be expected to interact with at least KCTD2 and KCTD5 (based on the literature), and perhaps KCTD9 or KCTD17 (considering the shared homology and experiments with Drosophila orthologs [16]). This would test our hypothesis that KCTD5 may form hetero-oligomeric structures with other KCTD family proteins.

**Figure 1:**
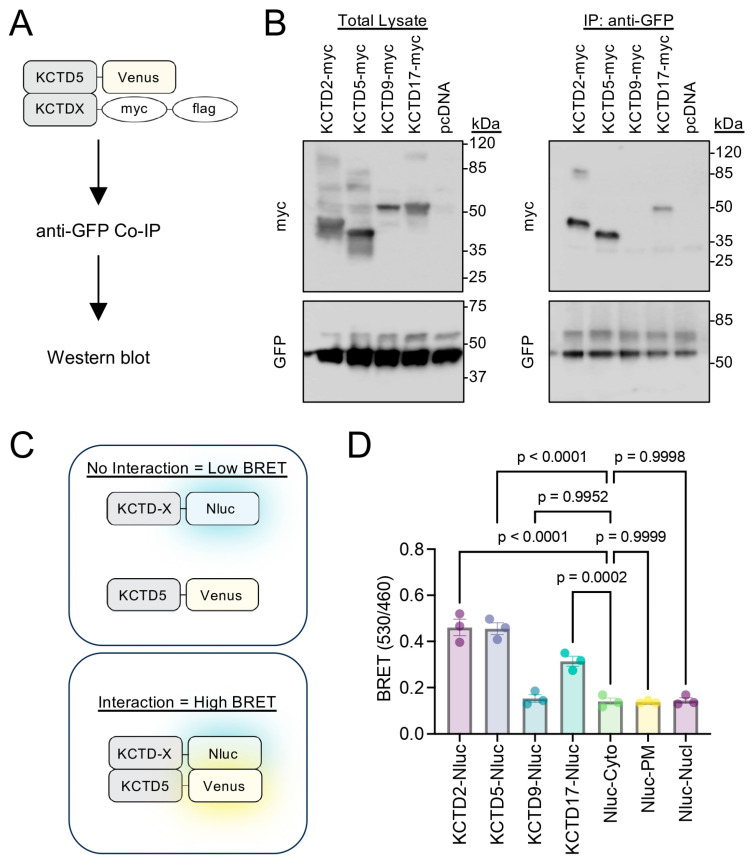
Characterization of cell-based approaches to examine KCTD5’s interaction with KCTD2, KCTD5, KCTD9, and KCTD17. (**A**) Scheme of the Co-IP experiment from HEK293 cells transfected with KCTD5-Venus and KCTD-myc-flag constructs in a 1:1 ratio for pulldown with the anti-GFP antibody. (**B**) IP of KCTD5-Venus from HEK293 cells with the anti-GFP antibody followed by probing for C-terminal myc-flag-tagged KCTD2, KCTD5, KCTD9, or KCTD17 with an anti-myc antibody. Representative blot from three independent experiments. (**C**) Scheme of the BRET assay to probe KCTD interactions between C-terminal Nanoluciferase (Nluc)-tagged KCTD (KCTD-Nluc) and C-terminal Venus-tagged KCTD5 in live cells. (**D**) Raw BRET ratio (530 nm Venus acceptor divided by 460 nm Nluc donor) from cells transfected with KCTD5-Venus and KCTD-Nluc-tagged (KCTD2, KCTD5, KCTD9, or KCTD17) constructs in a 1:1 ratio. Control cells include Nluc location in the cytosol (Nluc-Cyto), plasma membrane (Nluc-PM), and nucleus (Nluc-Nucl). Data plotted as mean ± SEM. n = 3 biological replicates per sample. One-way ANOVA, compared to the Nluc-Cyto sample with the Dunnett test was utilized for experimental comparisons between BRET samples, with *p*-values labeled for each group. Note that protein ladders from different companies were utilized, which also differ in their relative band size. Molecular mass from the protein ladder is presented in kilodalton (kDa). The predicted mass for each KCTD, based on UniProt ID, is listed in Supplementary Table S1.

First, we employed co-immunoprecipitation (Co-IP) from cells transiently transfected with C-terminal KCTD constructs: KCTD5-Venus and our KCTD of interest with a myc-flag tag (e.g., KCTD2-myc-flag) (Figure 1A). Cells were lysed following transfection to perform Co-IP with an anti-GFP antibody in order to pulldown KCTD5. We then performed Western blot analysis to detect the presence of KCTD5-Venus (anti-GFP antibody) and KCTD-myc-flag (anti-myc antibody) (Figure 1B). In agreement with previous literature [16,17], KCTD5 robustly interacted with both KCTD2 and KCTD5. Interestingly, KCTD5 also interacted with KCTD17 (although this appeared weaker) but not with KCTD9 (Figure 1B). To validate this result with an orthogonal approach in live cells, we developed a bioluminescence resonance energy transfer (BRET)-based assay. Cells were transfected with a C-terminal KCTD-Nanoluciferase construct (BRET donor, e.g., KCTD2-Nluc) and the same KCTD5-Venus construct (BRET acceptor) (Figure 1C). In this assay, a higher BRET signal would indicate proximity between KCTD proteins, thus suggesting a possible interaction. We previously validated that these KCTD-Nluc constructs yielded similar luminescence/expression in transfected cells [6]. To gauge the background signal, we also performed the BRET experiment with a cytosolic Nanoluciferase (Nluc-Cyto), a plasma membrane Nanoluciferase by fusion with the C-terminus of KRas (Nluc-PM), and a nuclear Nanoluciferase by fusion with the 3× N-terminal SV40 NLS (Nluc-Nucl). We observed a robust BRET signal in live cells from KCTD2, KCTD5, and KCTD17 but not from KCTD9 (Figure 1D). Moreover, the signal from KCTD9 was on par with the three Nanoluciferase controls (Nluc-Cyto, Nluc-PM, and Nluc-Nucl), thus representing the baseline non-interaction signal. Importantly, data from the IP and BRET delivered similar conclusions, which support our rationale that KCTD5 may indeed form complexes with additional members of the KCTD protein family. Altogether, these experiments further demonstrated two feasible cell-based methods to detect KCTD5’s interactions with other KCTD proteins.

### 2.2. Profiling KCTD5’s Interaction with Each KCTD Family Member

**Figure 2:**
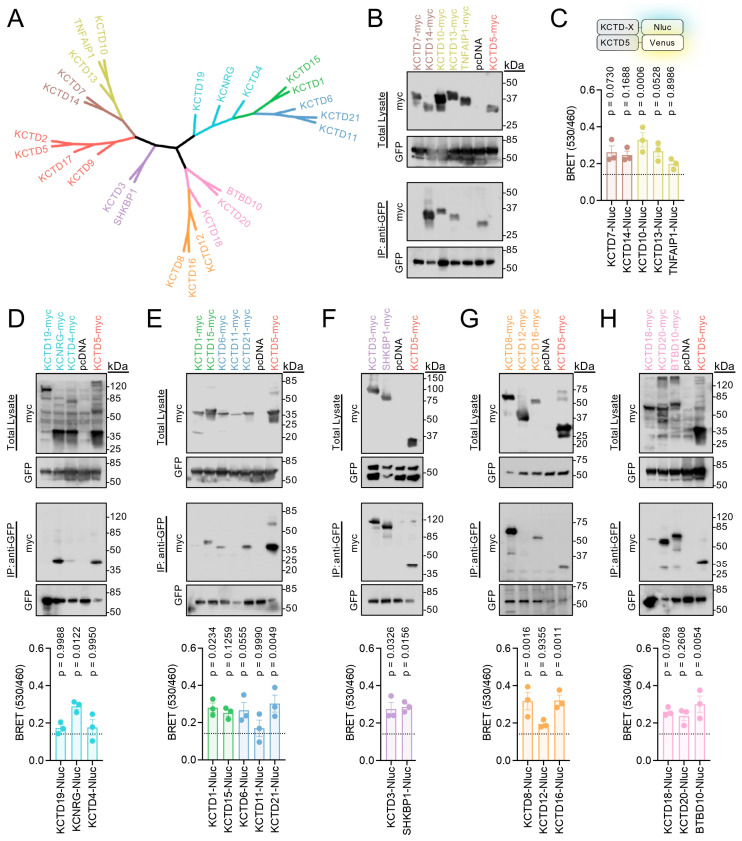
Profiling KCTD5’s interaction with the remaining KCTD family members. (**A**) Phylogenetic tree of human KCTD protein family. (**B**) IP of KCTD5-Venus from HEK293 cells with the anti-GFP antibody, followed by probing for C-terminal myc-flag-tagged KCTD7, KCTD14, KCTD10, KCTD13, or TNFAIP1 with an anti-myc antibody. Representative blot from three independent experiments. (**C**) Raw BRET ratio (530 nm Venus acceptor divided by 460 nm Nluc donor) from cells transfected with KCTD5-Venus and KCTD-Nluc-tagged (KCTD7, KCTD14, KCTD10, KCTD13, or TNFAIP1) constructs in a 1:1 ratio. (**D**) *top*: IP of KCTD5-Venus from HEK293 cells with the anti-GFP antibody, followed by probing for C-terminal myc-flag-tagged KCTD19, KCNRG, or KCTD4 with an anti-myc antibody. *bottom*: Raw BRET ratio (530 nm Venus acceptor divided by 460 nm Nluc donor) from cells transfected with KCTD5-Venus and KCTD-Nluc-tagged (KCTD19, KCNRG, or KCTD4) constructs in a 1:1 ratio. (**E**) *top*: IP of KCTD5-Venus from HEK293 cells with the anti-GFP antibody, followed by probing for C-terminal myc-flag-tagged KCTD1, KCTD15, KCTD6, KCTD11, or KCTD21 with an anti-myc antibody. *bottom*: Raw BRET ratio (530 nm Venus acceptor divided by 460 nm Nluc donor) from cells transfected with KCTD5-Venus and KCTD-Nluc-tagged (KCTD1, KCTD15, KCTD6, KCTD11, or KCTD21) constructs in a 1:1 ratio. (**F**) *top*: IP of KCTD5-Venus from HEK293 cells with the anti-GFP antibody, followed by probing for C-terminal myc-flag-tagged KCTD3 or SHKBP1 with an anti-myc antibody. *bottom*: Raw BRET ratio (530 nm Venus acceptor divided by 460 nm Nluc donor) from cells transfected with KCTD5-Venus and KCTD-Nluc-tagged (KCTD3 or SHKBP1) constructs in a 1:1 ratio. (**G**) *top*: IP of KCTD5-Venus from HEK293 cells with the anti-GFP antibody, followed by probing for C-terminal myc-flag-tagged KCTD8, KCTD12, or KCTD16 with an anti-myc antibody. *bottom*: Raw BRET ratio (530 nm Venus acceptor divided by 460 nm Nluc donor) from cells transfected with KCTD5-Venus and KCTD-Nluc-tagged (KCTD8, KCTD12, or KCTD16) constructs in a 1:1 ratio. (**H**) *top*: IP of KCTD5-Venus from HEK293 cells with the anti-GFP antibody, followed by probing for C-terminal myc-flag-tagged KCTD18, KCTD20, or BTBD10 with an anti-myc antibody. *bottom*: Raw BRET ratio (530 nm Venus acceptor divided by 460 nm Nluc donor) from cells transfected with KCTD5-Venus and KCTD-Nluc-tagged (KCTD18, KCTD20, or BTBD10) constructs in a 1:1 ratio. All Western blot data are representative blots from three independent experiments. All BRET data plotted as mean ± SEM, where the dotted line represents the average of experiments from Figure 1D with the Nluc-Cyto control plasmid. n = 3 biological replicates per sample. One-way ANOVA, compared to the Nluc-Cyto sample with the Dunnett test was utilized for experimental comparisons between BRET samples with *p*-values labeled for each group. Note that protein ladders from different companies were utilized, which also differ in their relative band size. Molecular mass from the protein ladder is presented in kilodalton (kDa). The predicted mass for each KCTD, based on UniProt ID, is listed in Supplementary Table S1.

We next utilized these two approaches to probe if KCTD5 interacted with the other members of the KCTD protein family. We assembled a phylogenetic tree from human KCTD protein sequences and organized experiments to compare similar clades [9] (Figure 2A). Beginning with IP of KCTD5-Venus and detection of myc-flag-tagged KCTD, we first observed that KCTDs within the same clade often exhibit differential affinity for KCTD5. For instance, KCTD7 and KCTD14 share 40% identity (BLAST result from Q96MP8 and Q9BQ13), and KCTD14 was in the Co-IP with KCTD5, whereas KCTD7 was not detected (Figure 2B). KCTD10 and KCTD13 similarly interacted with KCTD5, yet TNFAIP1 did not. Intriguingly, BRET results from KCTD5-Venus with KCTD-Nluc revealed a different profile (Figure 2C). Here, each KCTD yielded greater BRET than the bystander signal from cytosolic Nanoluciferase (Nluc-Cyto; dashed line on bar graph); however, only KCTD10 reached significance, while the other clades provided a consensus between assays. Both IP and BRET results showed that KCNRG, but not KCTD19 or KCTD4, interacted with KCTD5 (Figure 2D). In the case of KCTD1 and KCTD15 (80% identity from BLAST: Q719H9 and Q96SI1), we found only KCTD15 interacting with KCTD5 via IP yet KCTD1 exhibited a BRET signal significantly greater than the control (Figure 2E). Meanwhile KCTD6 and KCTD21 demonstrated interactions in both assays, whereas KCTD11 had no interaction with KCTD5 (Figure 2E). KCTD3 and SHKBP1 (68% identity from BLAST: Q9Y597 and Q8TBC3) followed the pattern whereupon they interacted with KCTD5 in both assays (Figure 2F). KCTD8 and KCTD16 also interacted with KCTD5 in IP and BRET while their clade-mate KCTD12 was not found to interact (Figure 2G). Finally, KCTD5 IP revealed a relatively weak interaction with KCTD18 and a strong interaction with KCTD20 and BTBD10 (Figure 2H). All three KCTDs also yielded a BRET signal greater than the control; however only BTBD10 was statistically significant. Collectively, this panel of data revealed the relative interaction strength between KCTD5 and each individual KCTD family member in both cell lysates and live cells.

### 2.3. Determination of Which Region on KCTD5 Facilitates Interaction with Other KCTD Family Proteins

**Figure 3:**
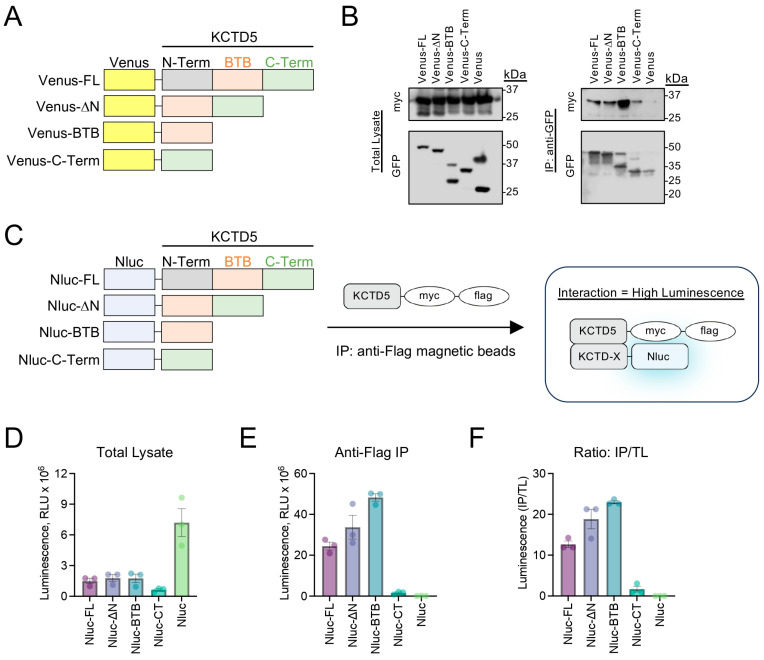
The KCTD5 BTB domain is necessary and sufficient for cellular KCTD5 homo-oligomers. (**A**) Scheme of N-terminal Venus-tagged KCTD5 constructs. (**B**) IP of Venus-KCTD5 fragments from HEK293 cells with the anti-GFP antibody, followed by probing for C-terminal myc-flag-tagged KCTD5 with an anti-myc antibody. Representative blot from three independent experiments. (**C**) Scheme of N-terminal Nluc-tagged KCTD5 constructs and experimental pipeline for IP with anti-flag-conjugated magnetic agarose beads for luminescence detection. (**D**) Total luminescence from total lysates of HEK293 cells expressing Nluc-KCTD5 fragments and KCTD5-myc-flag in a 1:1 ratio. (**E**) Total luminescence from anti-flag IP of HEK293 cells expressing Nluc-KCTD5 fragments and KCTD5-myc-flag in a 1:1 ratio. (**F**) Ratio of total lysate luminescence to anti-flag IP-luminescence of HEK293 cells expressing Nluc-KCTD5 fragments and KCTD5-myc-flag in a 1:1 ratio. All luminescence data plotted as mean ± SEM. n = 3 biological replicates per sample. Molecular mass from the protein ladder is presented in kilodalton (kDa).

The KCTD5 crystal structure reveals intermolecular contact points at the BTB–BTB domain interface [10]. This raises the question as to whether the BTB domain on KCTD5 is the universal motif that facilitates interactions with other KCTD proteins as well. Therefore, we utilized Co-IP to first verify KCTD5–KCTD5 interactions in the cellular environment. For this purpose, we generated the following KCTD5 constructs with Venus fused to the N-terminus: full-length KCTD5 (Venus-FL), deleted AA 1–39 on the KCTD5 N-terminus (Venus-ΔN), KCTD5 BTB domain AA 40–151 (Venus-BTB), and KCTD5 C-terminal AA 149–234 (Venus-CT) (Figure 3A). Detection with an anti-GFP antibody from HEK293 cell lysates indicated that each construct was well expressed following transient transfection alongside KCTD5-myc-flag (Figure 3B). Pulldown with the anti-GFP antibody revealed that Venus-KCTD5 formed a complex with KCTD5-myc-flag (Figure 3B). Moreover, deletion of the N-terminus did not prevent the KCTD5–KCTD5 interaction. The Venus-BTB qualitatively pulled down KCTD5-myc-flag much greater than Venus-FL, while the Venus-CT was able to pulldown a small fraction of KCTD5-myc-flag. Overall, the data was in agreement with the structural data that BTB largely facilitates the KCTD5–KCTD5 interaction [10].

We next sought to examine similar interaction profiles with other KCTD proteins and, therefore, developed a luminescence-based platform to increase the experimental throughput. We replaced the N-terminal Venus tag on each KCTD5 chimera with Nluc: Nluc-FL, Nluc-ΔN, Nluc-BTB, and Nluc-CT (Figure 3C). We then transiently transfected cells with Nluc-KCTD5 and KCTD5-myc-flag. In this pipeline the cells were lysed, luminescence was measured from the total lysate, Co-IP was performed with anti-flag-conjugated magnetic beads, and finally, luminescence was measured from the IP. Thus, pulldown was performed by KCTD5-myc-flag in these experiments, and interaction with Nluc-KCTD5 would be indicated by the luminescence signal. We first observed that luminescence readings were similar between each KCTD5 chimera in the total lysate and the Nluc-Cyto that was included as a control (Figure 3D). Readings from the IP revealed robust signals from Nluc-FL, Nluc-ΔN, and Nluc-BTB (Figure 3E), which matched the previous Western blot-based experiment. In order to adjust for potential variability between samples, we divided the IP-luminescence by the value of the total lysate (Figure 3F). The data again suggest that the KCTD5 BTB domain is necessary and sufficient to facilitate interactions between KCTD5 proteins. Moreover, the results validate the sensitivity of our IP-luminescence approach.

### 2.4. KCTD5 BTB and CT Domains Differentially Contribute to Interaction with KCTD Protein Family Members

**Figure 4:**
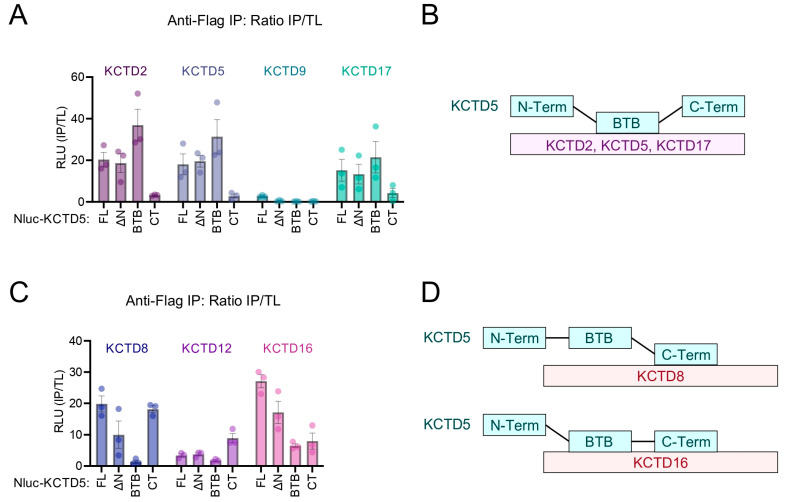
KCTD5 BTB and C-terminus differentially dictate hetero-oligomerization with KCTD proteins. (**A**) Ratio of total lysate luminescence to anti-flag IP-luminescence of HEK293 cell expression of Nluc-KCTD5 fragments and full-length KCTD (KCTD2, KCTD5, KCTD9, or KCTD17) constructs with a C-terminal myc-flag-tag in a 1:1 ratio. (**B**) Scheme summarizing KCTD5 BTB domain’s ability to interact with KCTD2, KCTD5, and KCTD17. (**C**) Ratio of total lysate luminescence to anti-flag IP-luminescence of HEK293 cell expression of Nluc-KCTD5 fragments and full-length KCTD (KCTD8, KCTD12, or KCTD16) constructs with a C-terminal myc-flag tag in a 1:1 ratio. (**D**) *top*: Scheme summarizing KCTD5 C-terminal domain ability to interact with KCTD8 and KCTD12. *bottom*: Scheme summarizing KCTD5 BTB and C-terminal domain ability to interact with KCTD16. All luminescence data plotted as mean ± SEM. n = 3 biological replicates per sample. RLU = relative luminescence unit.

We next utilized the IP-luminescence approach to probe regions of KCTD5 important for interactions with two distinct clades of the KCTD protein family. Considering their physiological importance to signal transduction, we first investigated KCTD2, KCTD5, KCTD9, and KCTD17 [7]. We also reasoned that existing data on the KCTD5–KCTD5 interaction in the literature as well as our previous figures would provide important internal controls in these experiments. Similar to Figure 1, we observed a strong luminescence signal from KCTD5 Nluc-FL with KCTD2-myc-flag, KCTD5-myc-flag, and KCTD17-myc-flag (Figure 4A). We also noticed the same pattern: the KCTD5 BTB domain was necessary and sufficient for interaction, whereas the KCTD5 CT was unable to interact (Figure 4B). In alignment with Figure 1, KCTD5 exhibited weaker complex formation with KCTD17 compared to with KCTD2 and KCTD5. Finally, we did not detect a KCTD5–KCTD9 interaction.

The next group we investigated was hetero-oligomerization with the KCTDs involved in shaping GABA_B_ signaling (KCTD8, KCTD12, and KCTD16) [13]. Similar to data in Figure 2, Nluc-FL KCTD5 formed a complex with KCTD8 and KCTD16 but not with KCTD12 (Figure 4C). Interestingly KCTD8 also interacted with KCTD5 Nluc-ΔN, not at all with KCTD5 BTB-Nluc, and the strongest with KCTD5 Nluc-CT, yet KCTD16 interacted equally as well with KCTD5 Nluc-BTB and Nluc-CT. On the other hand, KCTD12 was only able to interact with KCTD5 Nluc-CT. These data suggest that the KCTD5 C-terminus is important for interaction with KCTD8, KCTD12, and KCTD16 (Figure 4D). However, the interaction between full-length KCTD8 and KCTD5 does not seem to occur in the cellular environment. Altogether, these experiments reveal components of KCTD5 required for interaction with other KCTD family members.

## 3. Discussion

Our main finding was that KCTD5 broadly interacts with many, but not all, members of the KCTD protein family. We further determined that the KCTD5 BTB domain is capable of facilitating interactions with some KCTDs (2, 5, 16, 17), while the KCTD5 C-terminus is important for interactions with other KCTDs (8, 12, 16). Moreover, our results were enabled by the development of a Co-IP strategy, BRET-based cellular assay, and an IP-luminescence strategy, adding technical resources to advancing our understanding of KCTDs as well as conceptual insight.

The KCTD protein family is hallmarked by the presence of a BTB domain [2], a conserved motif of α-helices and a β-sheet [20], first identified in the *D. melanogaster* broad complex, tramtrack, and bric-à-brac genes [21,22]. The BTB domain functions as a protein–protein interaction module that typically facilitates self-assembly into multimers [23]. Crystal structures have appreciated that KCTD proteins typically assemble into such oligomers ranging from four to six subunits [4,10,24,25,26]. The crystal structure of KCTD5 revealed that pentameric formation was facilitated through BTB domain interactions [10]. Similarity in BTB domains within KCTD clades hints toward hetero-oligomeric complex assembly. Indeed, it was recently reported that KCTD5 forms a hetero-oligomer with KCTD2 [17], of which the overall identity between the two proteins is 82% (BLAST result, Q14681 and Q9NXV2). Moreover, the interaction with KCTD5 was found to be required for KCTD2’s recruitment of Cul3, thus conferring functional significance to the hetero-oligomer [17]. In a previous KCTD hetero-oligomer report it was demonstrated that all possible interactions occur between members of the clade consisting of KCTD8, KCTD12, and KCTD16 [14]. Functional significance here was demonstrated by experiments showing that the kinetics of GABA_B_ signaling are shaped by such hetero-oligomeric configurations [14].

Our data demonstrated that KCTD5 forms hetero-oligomers both within and beyond its clade and across the entire KCTD protein family. Our unbiased examination was performed utilizing two cell-based approaches: IP (lysed cells) and BRET (intact cells). The results between both approaches were largely in agreement with only a few exceptions. One reason for such discrepancy could be inherent to the technical aspects of the methodology. For instance, BRET-based experiments offer a high degree of sensitivity to detect interactions in live cells and yet rely on spatial positioning of donor/acceptor molecules. Considering the range of size across the KCTD family, approximately 20 to 120 kDa, it is possible that KCTD5 (~26 kDa) may not be optimally arranged for BRET with some KCTD proteins in our experimental design. On the other hand, Co-IP experiments were performed in a lysed environment that may not reflect the structural landscape of intact viable cells. On this basis, it may be expected to have a few outliers in regard to consistency in results between experimental approaches. Another possibility is that tag (i.e., myc-flag, Venus) placement (N- or C-terminus) could influence the activity of the protein. However in our previous work, we demonstrated that such effect did not occur in regard to full-length KCTD proteins [6]. Moreover, the BTB domain is the putative region involved in facilitating the majority of protein–protein interactions. This domain is generally more central in each KCTD sequence and is likely buffered from steric interference of fused tags. Nonetheless, we reinforced our results, in addition to mapping the precise region of KCTD5 required for hetero-oligomeric complex formation with an IP-luminescence strategy. Here, we focused on a handful of KCTDs (2, 5, 8, 12, 16, and 17) with established roles in neuronal signal transduction on both a cellular and in vivo level [7,27,28,29]. The KCTD5 BTB domain was found to be both necessary and sufficient for complex formation with KCTD2, KCTD5, and KCTD17. The results support structural data regarding the KCTD5 homo-pentamer [10] and suggest similar interactions may be involved with KCTD2 and KCTD17. Intriguingly, the interaction pattern was quite different with KCTD8, KCTD12, and KCTD16. Here, the KCTD5 C-terminus was necessary and sufficient for interaction with KCTD8. KCTD12 was not found to interact with KCTD5 in either Co-IP or BRET, yet the C-terminus of KCTD5 by itself was able to modestly interact with full-length KCTD8 in IP-luminescence experiments. Thus, the BTB domain of KCTD5 did not interact with KCTD8 or KCTD12. However, KCTD16 required both the BTB domain and C-terminus of KCTD5 for a full interaction. Therefore, KCTD5 hetero-oligomer formation is surprisingly not singularly dependent on the BTB domain. Importantly, this is not the first example of the KCTD C-terminus’ role in facilitating protein interactions. Trichoplein binds to the C-terminus of KCTD17 [30] whereas Gβγ interacts with the C-terminus of KCTD17 [6] and KCTD12 [4].

The positive interaction observed between the KCTD5 C-terminus and KCTD8/12/16 is further intriguing from a couple of viewpoints. The expression of KCTD5 C-terminal amino acids 154–234 have been reportedly unable to co-assemble and accumulate in inclusion bodies when stripped from the preceding portion of the protein [10,15]. One consideration from our experiments was that fusing either Venus or Nanoluciferase at the N-terminus sufficiently stabilized the KCTD5 C-terminus, at least in the case of the interaction between KCTD8/12/16 and C-terminal amino acids 149–234. Additionally, while the C-terminal sequence varies between KCTD5 and KCTD8/12/16 [6], the C-terminus of these proteins have significant structural similarity [11,31]. This may contribute to the intriguing interaction observed at the KCTD5 C-terminus in our data. As also mentioned, examples of KCTD C-terminal domains recognizing different partners have been well described [4,6,30]. Therefore, the KCTD5 C-terminus playing a role in KCTD hetero-oligomerization was unexpected. Nonetheless, our data hint toward a competitive dynamic interplay of the KCTD C-terminus between binding partners and other KCTD molecules. This concept may also be applied to the BTB domain, which has multifaceted roles in enabling KCTD oligomerization and substrate interactions (e.g., Cul3 and GABA_B_) [4,13,26,32,33,34,35,36]. Ultimately, KCTD5’s facilitation of hetero-oligomeric KCTD complexes brings into question the final form of such structures, with at least two possibilities. As KCTD multimers are frequently pentameric, one plausible scenario is pentameric formation of differing KCTD subunits. Such arrangement has been suggested between KCTD2 and 5; however, this remains to be experimentally validated [17]. On the other hand, it is worth considering that associations between KCTD pentamers could assemble higher-order complexes (e.g., decamers). Again, validation of such multimers would require further experimentation and analysis.

The findings here collectively suggest that KCTD5 may be intertwined in more aspects of cellular physiology than previously recognized. Hetero-oligomeric complex formation with various members of the KCTD family enables additional scaffolding opportunities via numerous possible KCTD subunit configurations. One implication may be the ability of KCTD5 to serve as a Cul3-Ubiquitin ligase adapter for cargo that does not directly bind KCTD5 itself but rather adjacent KCTDs in the hetero-oligomeric complex. This would add a layer of regulation to KCTD5’s control over substrate ubiquitination and turnover. In this manner, tissue-specific expression of KCTD5 with particular KCTD partners would enable precise control over the substrate protein lifetime. This could also foreseeably enable subcellular localization for pools of KCTD5, as necessary. We believe this could be one possible rationale for the redundancy in having more than two dozen KCTD proteins encoded in the human genome. Applying the concept more broadly, we suggest another exciting possibility, whereupon hetero-oligomeric complex formation exists between the vast majority of KCTD family proteins. It has already been established that KCTD12–KCTD16 form complexes in vivo, with profound influence on GABA_B_ signaling kinetics [14]. Therefore it is conceivable that heterogeneous interactions across the entire KCTD family may occur, thus adding unprecedented complexity toward the influence of KCTD on physiology and health. Although exciting, such systems-level interrogation of KCTD biology is beyond the scope of our work here, but it may nonetheless be a productive step towards future research efforts.

## 4. Materials and Methods

### 4.1. Bioinformatics

A KCTD phylogenetic tree was assembled by alignment of the following human KCTD protein UniProt IDs using the UniProt Align function: Q719H9, Q14681, Q9Y597, Q8WVF5, Q9NXV2, Q8NC69, Q96MP8, Q6ZWB6, Q7L273, Q9H3F6, Q693B1, Q96CX2, Q8WZ19, Q9BQ13, Q96SI1, Q68DU8, Q8N5Z5, Q6PI47, Q17RG1, Q7Z5Y7, Q4G0X4, Q9BSF8, Q8N5I3, Q8TBC3, and Q13829. The Newick notation was then rendered with phylo.io software (https://phylo.io/, accessed on 13 July 2023). Human sequences were utilized to reflect potential pathophysiological considerations and provide insight toward studying health in humans overall.

### 4.2. Cell Culture

Human embryonic kidney cells (Lenti-X HEK293T; Takara Bio #632180; San Jose, CA, USA) were grown in DMEM (Gibco #11995; Waltham, MA, USA) supplemented with 10% Fetalgro/FetalgroEX (RMBio; Missoula, MT, USA), minimum Eagle’s medium non-essential amino acids, 100 units/mL of penicillin, and 100 μg/mL of streptomycin. Cells were maintained in a 37 °C humidified incubator with 5% CO_2_. Plasmid transfection was performed with polyethylenimine (Polysciences #23966-100; Warrington, PA, USA) in OptiMEM (Gibco #11058021) on culture vessels or glass coverslips coated with PDL (Gibco #A38904). Medium was replaced with OptiPRO SFM (Gibco #12309019) approximately 24 h after transfection and cells were maintained for an additional 24 h before performing experiments.

### 4.3. Plasmids and Molecular Cloning

KCTD ORF constructs with C-terminal myc-flag tag, C-terminal Nanoluciferase (Nluc), and C-terminal Venus have previously been described [6]. The following additional Nluc constructs were generated by T4 ligation of codon-optimized Nluc (Twist Biosciences; San Francisco, CA, USA): cytosolic Nluc (Nluc-Cyto) in pTwist vector, plasma membrane Nluc (Nluc-PM) in pTwist vector with a C-terminal KRas membrane-targeting sequence [37], nuclear Nluc (Nluc-Nucl) in pTwist vector with N-terminal 3× NLS, and Nluc at the N-terminus of KCTD5 fragments (Nluc-FL, Nluc-ΔN, Nluc-BTB, and Nluc-CT) [6]. Venus at the N-terminus of KCTD5 fragments (Venus-FL, Venus-ΔN, Venus-BTB, and Venus-CT) was achieved by In-Fusion HD Cloning of KCTD5 fragments into digested mVenus-C1 (Addgene #27794; Watertown, NY, USA). Experiments with KCTD fragments utilized tags (Venus or Nluc) placed at the N-terminus as to not interfere with the region of interest during protein interaction studies, as similarly performed previously [6].

### 4.4. Co-Immunoprecipitation (Co-IP)

Cells were harvested by centrifugation into a pellet 48 h post-transfection, resuspended in ice-cold lysis buffer (PBX supplemented with 1% Triton-X, 150 mM NaCl, and Roche cOmplete protease inhibitor #11836170001; Indianapolis, IN, USA), and sonicated for 15 s at 30% power (FisherBrand #FB50110; Waltham, MA, USA). Lysates were centrifuged for 15 min at 12,000 rpm at +4 °C, whereupon the supernatant (total lysate) was transferred to a fresh tube. Then, 150 μg of the total lysate, as determined by the Pierce 660 Protein Assay Reagent (ThermoFisher #22660; Waltham, MA, USA), was subjected to Co-IP for a 2 h rotation at +4 °C in a total volume of 500 μL. For Western blot, 1 μg of an anti-GFP antibody (Santa Cruz; #sc-9996; Dallas, TX, USA) and 10 μL of Dynabeads Protein G (ThermoFisher #10004D) were mixed with the total lysate. Samples were then washed three times for 10 min before elution in SDS sample buffer by heating in a 37 °C water bath for 15 min.

### 4.5. Western Blot

Samples were diluted to the same concentration in SDS sample buffer and heated in a 37 °C water bath for 15 min. Total lysate utilized 10–15 μg of total protein and IP utilized 20 μL from the preparation detailed above. Samples were resolved by SDS polyacrylamide gel electrophoresis, transferred to PVDF membranes, and incubated with 5% dry non-fat milk (LabScientific #M0841; Danvers, MA, USA) in PBS containing 0.1% Tween-20 (PBST). Membranes were incubated with primary antibodies in 1% milk in PBST: mouse anti-GFP (Santa Cruz; #sc-9996), rabbit anti-GFP (ThermoFisher; #A6455), and rabbit anti-myc-tag (Cell Signaling Technology #2278; Danvers, MA, USA). Following washing in PBST, membranes were then incubated with secondary antibodies conjugated to horseradish peroxidase in 1% milk in PBST: mouse anti-rabbit (Jackson ImmunoResearch #211-032-171; West Grove, PA, USA) and goat anti-mouse (Jackson ImmunoResearch #115-035-174). Protein bands were visualized digitally with the KwikQuant Imager (Kindle Biosciences #D1001; Greenwich, CT, USA) following application of ECL reagent. The predicted mass for each KCTD, based on UniProt ID, is listed in Supplementary Table S1.

### 4.6. BRET Assay

Cells were transfected in a 1:1 ratio with KCTD5-Venus:KCTD-Nluc. After 24 h, the cells were washed once with PBS and then incubated in 5 mM of EDTA in PBS for 5 min to detach. Cells were then centrifuged at 5000× *g* for 5 min at room temperature and resuspended in BRET buffer (PBS containing 0.5 mM of MgCl_2_ and 0.1% glucose). Approximately 100,000 cells were transferred per well to a 96-well white opaque plate. Following a 5 min room-temperature incubation, an equal volume of freshly prepared 2× (42.2 μM) Hikarazine-108 Nanoluciferase substrate (21.1 μM final concentration) [38,39] was added to the BRET buffer. BRET measurements were then recorded in an opaque white-wall plate on a SpectaMax microplate reader (Molecular Devices; San Jose, CA, USA) for detection of Nluc (460 nm) and Venus (530 nm) emission. The BRET signal was calculated by dividing the acceptor (Venus) by the donor (Nluc) wavelength. All experiments were performed at room temperature.

### 4.7. IP-Luminescence Assay

Cells were transfected with Nluc-KCTD5 and KCTD-myc-flag constructs in a 1:1 ratio. After 24 h, the cells were washed once with PBS and then incubated in 5 mM of EDTA in PBS for 5 min to detach. Cells were then centrifuged at 5000× *g* for 5 min at room temperature, resuspended in ice-cold lysis buffer, and sonicated for 15 s at 30% power. Lysates were centrifuged for 10 min at 12,500 rpm at +4 °C and the supernatant was transferred to a new tube. Then, 3.3% of the sample was utilized for the total lysate luminescence measurements. The remaining 66.7% was subject to IP with 10 μL of Pierce Anti-DYKDDDDK Magnetic Agarose (ThermoFisher #A36797) for 2 h while rotating at +4 °C in a total volume of 500 μL. IP samples were then washed three times for 10 min before resuspension in BRET buffer. Samples (total lysate or IP) were incubated with Hikarazine-108 (4.22 μM final concentration) for 2 min in an opaque black-wall plate at room temperature and luminescence measurements were then recorded on a Mithras LB 940 microplate reader (Berthold Technologies; Oak Ridge, TN, USA).

### 4.8. Statistical Analysis

At least three biological replicates were performed for each experiment. Bar graphs represent the mean ± SEM overlaid with plots of each individual data point. Statistical analysis was performed with GraphPad Prism 9. One-way ANOVA, compared to the Nluc-Cyto sample, with the Dunnett test was utilized for experimental comparisons between BRET samples, with *p*-values labeled for each group. Statistical analysis was performed from all KCTD-Nluc with Nluc-Cyto for equivalent multiple comparisons.

## Data Availability

The data presented in this study are available upon request from the corresponding author.

## Supplementary Materials

The following supporting information can be downloaded at: https://www.mdpi.com/article/10.3390/ijms241814317/s1.
